# HPTAS: An Alignment-Free Haplotype Phasing Algorithm Focused on Allele-Specific Studies Using Transcriptome Data

**DOI:** 10.3390/ijms26125700

**Published:** 2025-06-13

**Authors:** Jianan Wang, Zhenyuan Sun, Guohua Wang, Yan Miao

**Affiliations:** 1 Faculty of Computing, Harbin Institute of Technology, No. 92 Xidazhi Street, Nangang District, Harbin 150001, China; 2 College of Computer and Control Engineering, Northeast Forestry University, No. 26 He Xing Road, Xiangfang District, Harbin 150040, China

**Keywords:** haplotype phasing, SNP, allele-specific expression, transcriptome, RNA-seq

## Abstract

Haplotype phasing refers to determining the haplotype sequences inherited from each parent in a diploid organism. It is a critical process for various downstream analyses, and numerous haplotype phasing methods for genomic single nucleotide polymorphisms (SNPs) have been developed. Allele-specific (AS) expression and alternative splicing play key roles in diverse biological processes. AS studies usually focus more on exonic SNPs, and multiple phased SNPs need to be combined to obtain better inferences. In this paper, we introduce an alignment-free algorithm HPTAS for haplotype phasing in AS studies. Instead of using sequence alignment to count the number of reads covering SNPs, HPTAS constructs a mapping structure from transcriptome annotations and SNPs and employs a k-mer-based approach to derive phasing counts from RNA-seq data. Using both next-generation sequencing (NGS) and the third-generation sequencing (TGS) NA12878 RNA-seq data and comparing with the most advanced algorithm in the field, we have demonstrated that HPTAS achieves high phasing accuracy and performance and that transcriptome data indeed facilitates the phasing of exonic SNPs. With the continued advancement of sequencing technology and the improvement in transcriptome annotations, HPTAS may serve as a foundation for future haplotype phasing methods.

## 1. Introduction

In diploid organisms, one of the two chromosomes is inherited from the mother and the other from the father. Haplotype phasing refers to identifying the chromosome haplotype inherited from each parent. The two haplotypes are highly consistent with each other except for a small portion of variants, in which single nucleotide polymorphism (SNP) is the most common type. Then the haplotype phasing problem can thus be framed as determining the SNP sequence on each haplotype [1].

Haplotype phasing is crucial for downstream analysis, such as genotype imputation [2,3,4] and testing for natural selection [5,6,7]. Allele-specific (AS) studies use SNPs to capture asymmetries in metrics between two alleles, such as gene expression and splicing patterns. This is usually caused by epigenetic modification and cis-acting genetic variation [8], and these are involved in many essential biological mechanisms, such as random X-chromosome inactivation [9], genomic imprinting [10], and cis-regulation. With the advancement of high-throughput sequencing technologies, AS studies can extract insights from RNA-seq data and use computational methods to identify allele-specific differences [11,12,13,14,15]. Haplotype phasing can integrate information from multiple SNPs to improve the statistical power of estimates [16,17], thereby playing a key role in AS studies. In addition, the long reads in third-generation sequencing (TGS) data can span more distant SNPs compared to next-generation sequencing (NGS), potentially leading to more accurate phasing results [18,19].

A wide range of methods and algorithms have been developed to address the phasing problem. Haplotype phasing methods typically rely on modeling haplotype frequencies and estimating the probability of any given haplotype configuration based on a statistical model, followed by selecting the most likely configuration [20,21]. The statistical models contain Clark’s algorithm, the EM algorithm, coalescent-based methods, and hidden Markov models, which are used by PHASE [22], fastPHASE [23], MACH [24], IMPUTE2 [25], and BEAGLE [26]. Different methods also focus on different dimensions of the haplotype phasing problem, including population-based statistical phasing methods [23,24,25], solutions tailored for polyploid organisms [27,28,29], and algorithms for long reads, including FALCON-Phase [30] and DCHap [31].

For AS studies, individual sequencing data are used because mRNA expression on both alleles is measured in the same cellular context, minimizing the influence of environmental or trans-acting factors, facilitating the analysis of cis-regulatory elements and epigenetic variations. We present HPTAS, an algorithm and software for haplotype phasing from RNA-seq data using existing SNP and transcriptome information for allele-specific analysis. It uses a k-mer-based alignment-free approach for phasing and supports both NGS and TGS data. HPTAS is freely available as a PyPI Python package. The source codes of HPTAS are freely available at https://github.com/wjnjlcn/hptas, accessed on 10 June 2025.

## 2. Results and Discussion

### 2.1. Evaluating the Performance of HPTAS

Most existing tools perform haplotype phasing on DNA-seq data. However, genomic and transcriptomic data differ significantly in structural features and SNP density. In order to verify the performance of HPTAS, we used HapTree-X, the best tool for haplotype phasing based on RNA-seq data, for comparison [32]. Since HapTree-X does not support TGS sequencing data, we compared the type 1 results (accurate phased results) identified by the two tools on the PE dataset and performed a comprehensive test comparison on chromosomes 1, 5, 11, 15, and 21.

We screened the results with a supporting number of not less than three as the valid HapTree-X results. In HPTAS, we used mean>0.75 and 95% HDI lowerbound>0.5 or mean<0.25 and 95% HDI upperbound<0.5 to screen the valid results, which also corresponds to the number of reads being not less than three.

As shown in Table 1, although HapTree-X identified more phasing results, its accuracy was generally lower than that of HPTAS. This is because HapTree-X directly selected the results with a larger number of supporting reads as phasing results, and the results may be inaccurate when the number of reads is small.

**Figure 1:**
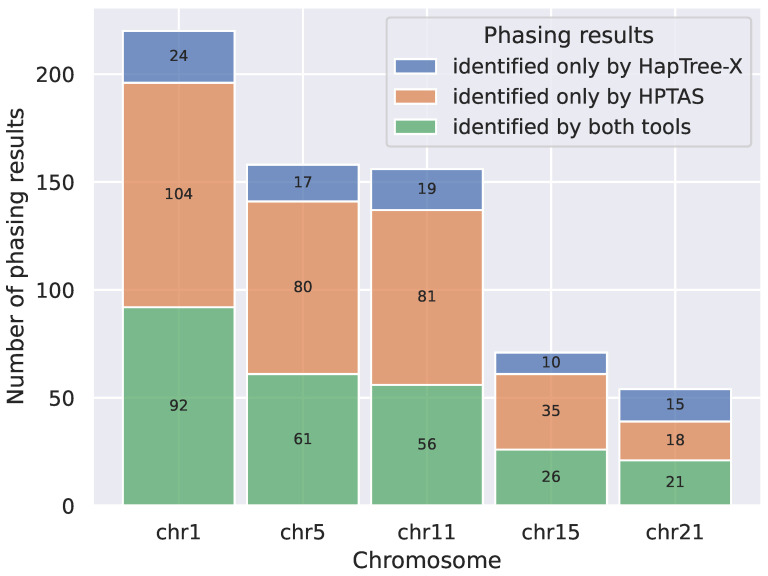
Overlap between HPTAS and HapTree-X type 1 phasing results on different chromosomes.

We then analyzed the overlap between HPTAS and HapTree-X type 1 phasing results on different chromosomes. As shown in Figure 1, on each chromosome, HPTAS identified the majority of results reported by HapTree-X, and the total number of identifications exceeded that of HapTree-X, demonstrating the performance advantage of HPTAS.

**Figure 2:**
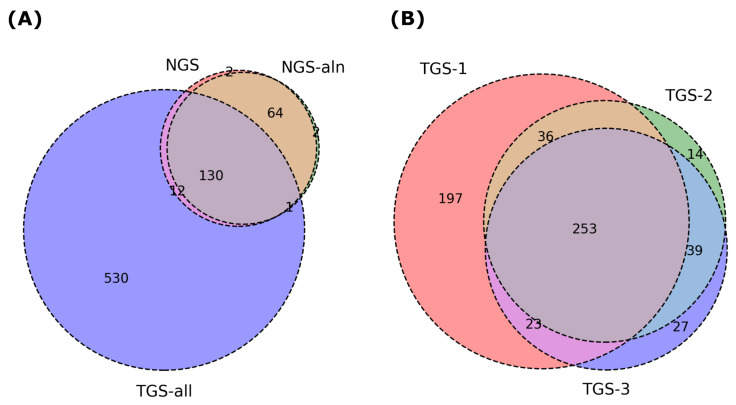
Haplotype phasing results identified by HPTAS using different datasets. (**A**) Comparison of phasing results between the NGS and combined TGS datasets. (**B**) Comparison of phasing results for three TGS datasets.

We then conducted a statistical analysis of the haplotype phasing results across the datasets. Table 2 summarizes the number of SNP pairs generated by HPTAS for each dataset. These results were compared, and Venn diagrams were constructed (Figure 2). The results of NGS and NGS-aln show high consistency, indicating the reliability of our alignment-free method and the discriminatory power of sequences with k=32. We also examined the phasing counts corresponding to the unique phasing results in the NGS-aln dataset and found that they were caused by the low and incomplete mapping counts due to the close distance of the SNPs during the sequencing alignment.

The high consistency of the two biological replicates TGS-2 and TGS-3 demonstrates the robustness of HPTAS. Overall, the results of the three TGS datasets are comparable. Although TGS-1 has a lower sequencing depth, it has identified more phasing results, suggesting the instability and randomness of third-generation sequencing data. In actual research, high coverage or combination with second-generation reads is still necessary.

**Figure 3:**
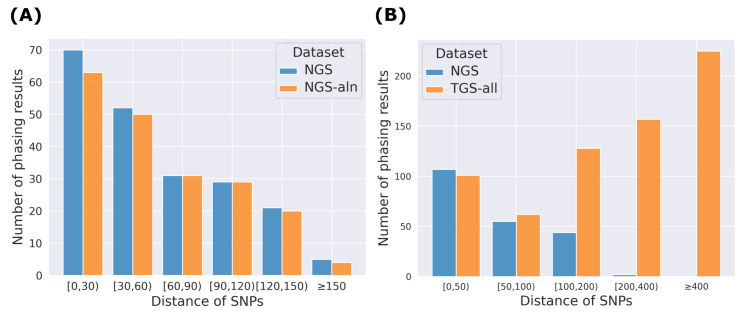
The relationship between the number of phasing results and SNP distances. (**A**) Comparison of relationships between NGS and NGS-aln datasets. (**B**) Comparison of relationships between NGS and combined TGS datasets.

In addition, we analyzed the relationship between the number of phasing results and SNP distances, all of which were transcriptome distances of SNP pairs involved in each phasing result (Figure 3). For NGS and NGS-aln, mapping-based methods may have difficulty or lack flexibility to handle SNPs that are very close to each other, resulting in missing phasing counts, which indicates the advantage of the alignment-free k-mer-based approach used in HPTAS. A Comparison of NGS and TGS-all showed that when the SNP distance is between 0 and 100, the number of phasing results identified by NGS and TGS-all is comparable. At larger SNP distances, NGS identifies fewer phasing results than TGS-all.

### 2.2. Advantages of Using RNA-Seq for Haplotype Phasing of Exonic SNPs

Among all 117,975 phased SNPs used in our experiments, 4111 were located on the exons of genes on chromosome 1. Of the genes, 742 genes had only one phased SNP, whereas 932 genes had multiple SNPs. In total, we performed haplotype phasing on 3180 SNP pairs, and we summarize the detailed statistical characteristics in Table 3. The SNP distance on the transcriptome is substantially smaller than that on the genome, indicating that RNA-seq can enhance haplotype phasing on exonic SNPs.

**Figure 4:**
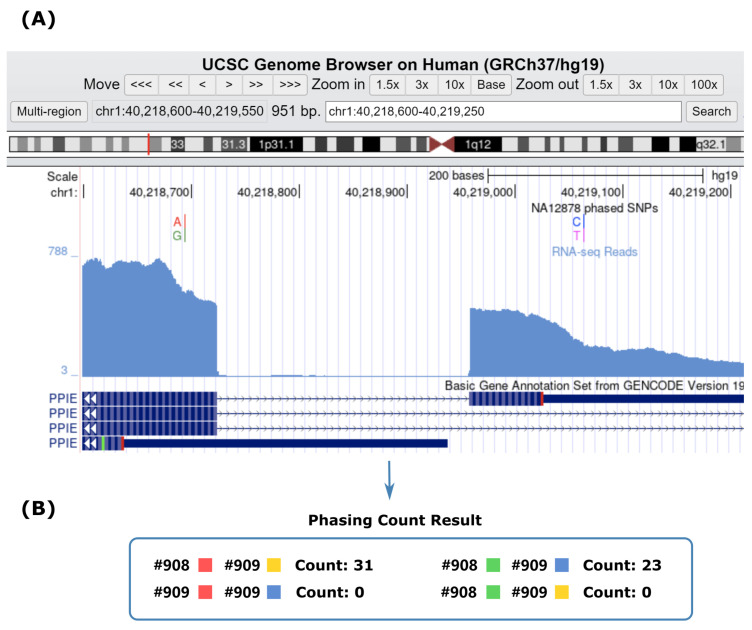
A detailed example of phasing count result demonstrating the benefits of haplotype phasing using RNA-seq. (**A**) Genome browser screenshot showing the SNP pair information along with nearby sequencing alignments and gene annotations. (**B**) Phasing count results of each possible haplotype combination.

Figure 4 presents a detailed example demonstrating the benefits of haplotype phasing for exonic SNPs using RNA-seq data. The screenshot of the Genome browser shows the SNP pair chr1: 40218695 (A/G) and chr1: 40219065 (C/T) on gene ENSG00000084072.12 (PPIE), as well as the read coverage of the NGS dataset. Although the SNPs are 370 bp apart on the human genome, they are only 136 bp apart on the first transcript of the gene. Therefore, even though the read length of the paired-end sequencing data is only 76 bp and the average insert size is 152.45 bp, sufficient phasing counts were obtained for this SNP pair. Here, a total of 54 type 1 phasing counts (31 AC and 23 GT) against 0 type 2 phasing counts were obtained, yielding a highly accurate haplotype phasing result. These results suggest that for AS studies focusing on exonic SNPs, RNA-seq can indeed facilitate haplotype phasing.

### 2.3. Discussion

For the haplotype phasing problem, typically, the first step in processing sequencing data is sequence alignment, which uses a specific algorithm to map the reads to a reference genome to determine their location [33,34]; then, counting the reads becomes a simple task. However, there are several problems with this alignment-based approach. First, locating each read from an organism with a huge genome is computationally expensive. In addition, for the haplotype phasing problem, reads are required to cover more than one SNP, which is contrary to the accuracy required for alignment. Although the sequence aligner can adjust its parameters to reduce sensitivity, its tolerance for errors is also increased, and this setting is not targeted at this specific problem. Moreover, RNA-seq data used for AS studies are aligned to the transcriptome rather than the genome, and each gene can contain dozens of transcripts, which makes the alignment process complex and time-consuming and also reduces its ability to process SNP information.

In addition, existing methods usually use whole-genome sequencing data for analysis, while AS studies focus more on exonic SNPs. Using the genome for haplotype phasing will obviously yield a greater number of phasing results overall, as it considers not only exonic SNPs but also SNPs on intron or regulatory regions, but using the transcriptome will bring additional benefits. Although two SNPs are far apart on the genome, they may be quite close on one of the RNA transcripts of the corresponding gene because there may be several long introns between them. In this case, RNA-seq obviously has more reads that may cover the pair of SNPs, thus providing more useful information for phasing.

In this paper, we proposed HPTAS, an algorithm for haplotype phasing based on an alignment-free approach using transcriptome data, and its accuracy has been verified experimentally using type 1 phasing results given already known phased SNPs of the NA12878 individual. However, the type 2 results (inconsistent results) do not necessarily mean erroneous phasing results; as we examined, a large number of inconsistent results had highly reliable type 2 phasing results and a portion of the results had a large number of both type 1 and 2 phasing counts, indicating that the pair of SNPs actually contained one homozygous site and one heterozygous site. Since the phased SNP of NA12878 used in our experiments actually comes from the genomic sequence, we can infer that the above situations are caused by sequence mutation during transcription.

It should also be mentioned that haplotype phasing results are sensitive to the SNPs used, and in our experiments, we only used the already phased SNPs to verify the accuracy of HPTAS. In actual applications, a more common configuration containing much more SNPs will be used. More SNPs will result in a greater SNP density on the transcriptome, leading to more available phasing results.

As shown in the experiment results, the high depth of the next-generation reads is more conducive to processing SNPs with closer distances, while the long read length in the third-generation data is more conducive to processing SNPs with farther distances, suggesting that each sequencing technology has its own advantages and that hybrid use is important in haplotype phasing.

HPTAS has two main limitations. The first is the SNP density, which affects all haplotype phasing algorithms. It is easy to see that the greater the SNP density, the more likely it is that there will be more reads spanning the corresponding SNP sites, thus giving more accurate phasing results. This depends on the accuracy and sensitivity of a good upstream SNP calling algorithm. The second is HPTAS’s requirement for the integrity of the transcriptome. The more accurate and complete the annotations of genes and transcripts are, the more accurate it can be when extracting k-mers of SNPs close to splicing sites. However, the transcriptome annotations of many species are still incomplete, so it may require more heuristic and adaptive improvements.

## 3. Materials and Methods

### 3.1. K-Mer-Based Alignment-Free Algorithm for Haplotype Phasing

Sequencing-based haplotype phasing methods fundamentally rely on reads to connect the heterozygous SNPs to be phased. We referred to a specific nucleotide allele of a heterozygous SNP as an SNP allele. If a read covered two SNPs and contained the corresponding *SNP alleles*, then this SNP allele pair may be inferred to belong to the same haplotype. However, errors in reads will mislead the results; the greater the number of reads supporting an SNP allele pair, the more reliable the phasing result. Therefore, the main task of haplotype phasing is to count the number of reads in the sequencing data supporting each possible SNP allele sequence.

Here, we introduced an alignment-free algorithm, HPTAS, which employs a k-mer-based approach to solve the haplotype phasing problem. K-mers are substrings of fixed length *k* found in biological sequences, and they are often used for genome assembly, sequence error correction [35], and variant calling [36,37]. HPTAS uses k-mers containing SNPs to build a mapping data structure, splits each read into k-mers to determine the corresponding SNP alleles, and it also uses k-mer frequencies to filter errors.

**Figure 5:**
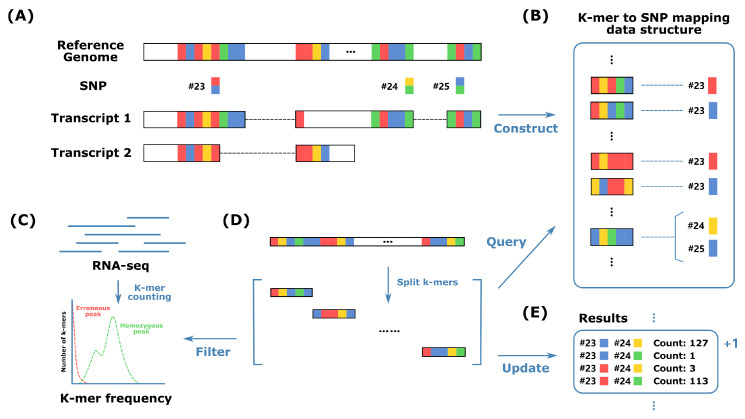
Haplotype phasing process using HPTAS. (**A**) Extract k-mers for each SNP across all transcripts. (**B**) Construct mapping data structures using k-mers and their corresponding SNP alleles. (**C**) Error k-mers were filtered out using k-mer frequencies counted from RNA-seq data. (**D**) Reads are split into k-mers to query for phasing results. (**E**) Phasing counts are updated based on retrieved results.

HPTAS started by analyzing existing transcriptome annotations and SNPs. Transcriptome annotations include every known transcript for each gene, and high completeness is desirable because RNA-seq reads can come from any transcript. For each gene, only SNPs located in exons of at least one transcript of the gene were used, even if it represented the sole SNP on a transcript, which may not seem to be helpful for phasing. However, the transcriptome annotation may be incomplete, and unknown transcripts containing these SNPs along with others may exist. HPTAS then extracted k-mers that covered at least one SNP allele. It used a sliding window of length *k* to split the contiguous k-mers around the known SNP sites (Figure 5A). This process was repeated for each transcript, because SNPs close to splicing junctions may have different k-mers between transcripts.

As shown in Figure 5B, HPTAS constructed a mapping structure using the k-mers and their corresponding SNPs. For each k-mer, it substituted the nucleotide base at the SNP site to each SNP allele and mapped each constructed k-mer to its corresponding SNP alleles for query. It should be noted that a k-mer can map to more than one SNP allele if it covers multiple SNP sites. In the above discussion, we assumed that the SNP information is for the specific individual. This process can be extended to the scenario where the SNPs of the organism are used. In this case, only the genomic location is relevant, and the SNP alleles can be assumed to be all possible nucleotides, namely A, C, G, and T, and they can be used to construct k-mers, respectively. However, if SNPs are not used at all, k-mers would be extracted from all transcript sequences, and the determination of SNPs would be achieved in the online processing of the read sequences.

HPTAS subsequently analyzed each read in the RNA-seq data (Figure 5D). Since the mapping structure already contained all possible k-mers covering SNPs, it did not need to extract k-mers in a window sliding approach. Instead, it extracted adjacent k-mers sequentially from the start to the end, and if the read length was not a multiple of k, it extracted an additional k-mer from the tail. K-mer frequencies were counted directly from the RNA-seq data, which can be a good indicator of erroneous k-mers, which have very low frequencies and were eliminated from the phasing process (Figure 5C).

Algorithm 1 illustrates the method for extracting k-mers from sequencing reads. Each k-mer was used to query the mapping structure to retrieve its corresponding SNP allele, and the results were merged into the query result of the entire read. The phasing counts of the corresponding SNP allele pairs were then updated based on the results (Figure 5E). For paired-end RNA-seq data, each read pair was treated as a single read. Additionally, since a read sequence can come from either strand, if the query did not return a valid result, its reverse complement sequence was analyzed again. Following the previous discussion, in the case where SNPs were not used, the hamming neighbors of each k-mer would need to be used for queries and the SNP position and possible phasing results would need to be directly reported.
**Algorithm 1** Query the codes of SNP alleles covered by a specific sequencing read.
    **Input:** sequencing read R of length *L*, main hash map *H* and alternative hash map $H^{\prime }$,      and pre-defined k-mer length *k*

    **Output:** set of SNP allele codes *S*
  1:**procedure** *MapRead*(*R*)  2:    S←∅  3:    N←L/k  4:    **for** n←1toN **do**  5:          i←(n−1)*k  6:          M←R[i+1…i+k]  7:          MapKmer(M,S)  8:    **end for**  9:    **if** Lmodk≠0 **then**10:        M←R[L−k+1…L]11:        MapKmer(M,S)12:    **end if**13:**end procedure**14:**procedure** *MapKmer*(M,S)15:       v←H{M}16:       s←v≫117:       S←S∪{s}18:    **if** (v&1)=1 **then**19:          $S\leftarrow S\cup H^{\prime }\left\{M\right\}$20:    **end if**21:**end procedure**


HPTAS reported SNPs in pairs for phasing count results. For multiple SNPs within a gene, phasing count results were reported for every two SNPs that were adjacent in order of genomic location. This approach was adopted because an increase in the number of SNPs per gene exponentially raised the possible haplotype combinations, thereby increasing the complexity of the phasing process.

### 3.2. Internal Structure of HPTAS

HPTAS stored all possible k-mers covering SNPs in a mapping structure maintained in memory during phasing, so the keys and values should be encoded appropriately to optimize memory usage. The encoding of k-mers is intuitive and conventional; we encoded *A* as 00, *C* as 01, *G* as 10, and *T* as 11, with the base codes concatenated into a single unsigned integer. A k-mer length of 32 has been shown in previous studies to provide ideal identifiability, and such k-mers fit neatly into a 64-bit unsigned integer, which aligns well with modern computer architectures.

**Figure 6:**
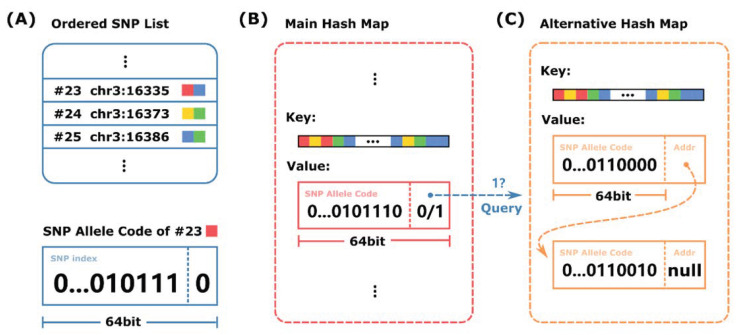
The internal structure of HPTAS. (**A**) The SNPs used for phasing are sorted and stored separately along with their metadata, and then SNP allele codes are generated based on the new index. (**B**) HPTAS uses two hash maps to implement the mapping structure to reduce memory waste. The main hash map contains the first SNP allele covered by each k-mer. (**C**) The alternative hash map contains all the remaining SNP alleles for the same k-mer key.

Each value in the mapping structure must identify both the SNP and the nucleotide base represented by the SNP allele. SNPs contain extensive metadata and are identified by their SNP ID string field. Instead of using the SNP ID, we sorted all the SNPs used in the phasing process into a specific order (e.g., by genomic position) and stored them separately for later querying. Then, we used the index of the list as the SNP identifier (Figure 6A).

The mapping structure was implemented using a hash map data structure due to its fast lookup time; however, this design may lead to potential memory inefficiencies in the context of phasing. The inefficiency arises when a k-mer covers multiple SNPs, which only occurs when two SNPs are less than 31 bp apart, a relatively rare scenario. To account for this albeit small possibility, we added an additional memory address field to each key value hash map item if the SNP alleles are stored via a linked list. Even if SNP alleles are stored in arrays and hash map items are dynamically allocated, an additional integer is still required to record the array’s length.

As shown in Figure 6B,C, HPTAS implemented two hash maps to solve the problem. The main hash map contained all possible k-mers covering SNPs along with the first SNP allele covered by each k-mer. Any additional SNPs covered by the same k-mer could be retrieved from an alternative hash map using the same key (Algorithm 1). Given the small number of entries, the alternative hash map could be implemented using a standard approach. Additional SNP alleles may also be stored in alternative data structures (e.g., trees) to further reduce memory usage without significantly affecting search performance.

### 3.3. Statistical Analysis

The primary algorithmic task of the haplotype phasing problem addressed in this paper is to count the number of reads covering each SNP pair, after which HPTAS applies statistical methods to quantify the phasing results.

For a pair of SNPs, we used $C_{i,j}$ to denote the number of reads that covered the pair and supported the i−th and j−th alleles of the two SNPs, respectively, where i,j∈{1,2}. For two SNPs there were only two possible haplotype phasing results. There were only two possible haplotype phasing results for two SNPs; for example, SNPs A/T and C/G can be phased as AC (TG) or AG (TC). We defined them as type 1 and type 2 haplotype phasing results, respectively. Then the number of type 1 phasing counts *H* is computed as

(1) $$H=\sum_{k=1}^{2}C_{k,k}$$

And the total number of reads covering the SNP pair *N* is

(2) $$N=\sum_{j=1}^{2}\sum_{i=1}^{2}C_{i,j}$$

We assumed that if θ is the probability of the type 1 phasing result, then the probability of the type 2 result is 1−θ. HPTAS used the Bayesian method, treating θ as a random variable and inferring its posterior distribution. *H* was modeled as a binominal distribution given θ, and we chose the beta distribution as the prior distribution due to its flexibility. Then, we derived the posterior with Bayes’ theorem:

(3) P(θ|H)∝θ(α−1)(1−θ)(β−1)B(α,β)NHθH(1−θ)N−H

Because for the haplotype phasing problem, a particular haplotype should be unambiguously phased, we expect θ to be close to 0 or 1, and accordingly, we chose α=0.5 and β=0.5 for the prior beta distribution to reflect this tendency.

HPTAS employed the stan package to construct the Bayesian model and used the MCMC (Markov chain Monte Carlo) method to generate posterior samples [38], which were used to construct the posterior distribution to infer the Bayesian estimate of θ, and the HDI (highest density interval) was used to determine the accuracy of the inference.

### 3.4. Datasets for Testing Haplotype Phasing

HPTAS supported both next-generation and third-generation RNA-seq data. NGS datasets usually have high sequencing depth and are readily available because the technology has been developed over many years. TGS datasets tend to have long sequencing read lengths, which are naturally suitable for haplotype phasing. To test the performance of HPTAS, we obtained four RNA-seq datasets of individual NA12878 from the ENCODE project, both NGS and TGS. The characteristics of the datasets are summarized in Table 4. The NGS dataset was sequenced in paired-end data, which has better phasing ability than single-end data, with an average inferred insert size of 152.45. Due to the lower sequencing depth compared to NGS, we included three TGS datasets containing a group of biological replicates.

The human transcriptome annotation used in our tests was GENCODE v19 [39], and we tested HPTAS on each dataset on chromosome 1, with 5363 genes. The SNPs of individual NA12878 were obtained from a previously published study [40], and we used the already phased SNPs to validate the phasing accuracy of HPTAS.

We performed haplotype phasing on each dataset to evaluate the performance of HPTAS. Furthermore, we added two procedures to supplement additional results. The first was the phasing result using sequence alignment of the paired-end NGS dataset for comparison with our alignment-free method, and the other was the combined phasing result using three TGS datasets to form a moderate sequencing depth for comparison with the high-depth NGS dataset. For all experiments, we eliminated k-mers that appeared only once as erroneous k-mers.

In the VCF file containing the NA12878 phased SNPs, the genotype “0|1” or “1|0” both indicate that the corresponding SNP has been phased. “0|1” means that the reference base is located in haplotype block 0 and the alternative base is located in haplotype block 1. Therefore, for SNPs with a genotype of “1|0”, we inverted the reference base and the alternative base in the SNP line to make the genotype consistent with “0|1”. The newly generated SNP profile was then used to evaluate the accuracy of HPTAS, and the above dataset was used for evaluation. Since we recorded the SNPs in the same order as the provided phased SNPs, the phasing results of type 1 indicate that the phasing results are consistent with the provided phased SNPs, that is, accurate phasing results.

## 4. Conclusions

In this paper, we proposed an algorithm and software tool, HPTAS (https://github.com/wjnjlcn/hptas), designed for the haplotype phasing of exonic SNPs, which is important for allele-specific studies. We demonstrated that the k-mer-based, alignment-free strategy used in HPTAS provides good phasing performance and accuracy. We also showed that RNA-seq data can facilitate the phasing of exonic SNPs when guided by transcriptome annotations rather than genomic sequences. Although relatively complete transcriptome annotations are required, often a limitation for non-human organisms, we proposed heuristic strategies to mitigate this issue. As sequencing technology evolves, HPTAS is expected to adapt to new data types and inspire future haplotype phasing methods.

## Figures and Tables

**Table 1:** Haplotype phasing results identified by HPTAS and HapTree-X.

	Chr 1	Chr 5	Chr 11	Chr 15	Chr 21
Exonic SNPs on the chromosome	4682	2828	3226	1720	822
Valid phasing results for HapTree-X	230	165	169	76	51
Type 1 phasing results for HapTree-X	116 (50.4%)	78 (47.3%)	75 (44.4%)	36 (47.4%)	36 (70.6%)
Valid phasing results for HPTAS	208	148	140	62	43
Type 1 phasing results for HPTAS	196 (94.2%)	141 (95.3%)	137 (97.9%)	61 (98.4%)	39 (90.7%)

**Table 2:** Haplotype phasing results for each dataset.

	NGS	NGS-Aln	TGS1	TGS2	TGS-3	TGS-All
Phasing counts valid for inference	245	225	630	429	435	804
Valid phasing results	208	197	509	342	342	673
Type 1 phasing results	196	194	493	329	329	657
Type 2 phasing results	12	3	16	13	13	16

**Table 3:** Statistical characteristics of the phased SNPs used in the experiment.

	Minimum	Maximum	Average
Number of SNPs located on gene	2	37	4.41
SNP distance on genome	1	1,434,890	7613.01
SNP distance on transcriptome	1	32,415	546.13

**Table 4:** RNA-seq dataset used to test the performance of HPTAS.

Dataset Name	Accession Number	Number of Bases	Number of Reads/Pairs	Sequencing Type	Read Length	
					Min	Max
NGS	ENCSR000COQ	17,917,200,640	117,876,320	paired-end	76	76
TGS-1	ENCSR962BVU	2,687,061,689	1,673,768	single-end	50	10,743
TGS-2	ENCSR838WFC	3,432,469,562	2,137,168	single-end, replicate 1	50	12,755
TGS-3	ENCSR838WFC	3,840,259,898	2,538,701	single-end, replicate 2	50	6456

## Data Availability

HPTAS can be installed as a Python package via pip; the source codes are freely available at https://github.com/wjnjlcn/hptas.
